# Promoting Help-Seeking in Response to Symptoms amongst Primary Care Patients at High Risk of Lung Cancer: A Mixed Method Study

**DOI:** 10.1371/journal.pone.0165677

**Published:** 2016-11-04

**Authors:** Richard Wagland, Lucy Brindle, Sean Ewings, Elizabeth James, Mike Moore, Carol Rivas, Ana Ibanez Esqueda, Jessica Corner

**Affiliations:** 1 Faculty of Health Sciences, University of Southampton, Highfield, Southampton SO17 1BJ, United Kingdom; 2 Southampton Statistical Sciences Research Institute, Faculty of Social, Human and Mathematical Sciences, Highfield, University of Southampton, Southampton, United Kingdom; 3 Faculty of Medicine, University of Southampton, Highfield, Southampton SO17 1BJ, United Kingdom; 4 Executive Office, The Nottingham University, University Park, Nottingham, NG7 2RD, United Kingdom; BIDMC, UNITED STATES

## Abstract

**Background:**

Lung cancer symptoms are vague and difficult to detect. Interventions are needed to promote early diagnosis, however health services are already pressurised. This study explored symptomology and help-seeking behaviours of primary care patients at ‘high-risk’ of lung cancer (≥50 years old, recent smoking history), to inform targeted interventions.

**Methods:**

Mixed method study with patients at eight general practitioner (GP) practices across south England. Study incorporated: postal symptom questionnaire; clinical records review of participant consultation behaviour 12 months pre- and post-questionnaire; qualitative participant interviews (n = 38) with a purposive sample.

**Results:**

A small, clinically relevant group (n = 61/908, 6.7%) of primary care patients was identified who, despite reporting potential symptoms of lung cancer in questionnaires, had not consulted a GP ≥12 months. Of nine symptoms associated with lung cancer, 53.4% (629/1172) of total respondents reported ≥1, and 35% (411/1172) reported ≥2. Most participants (77.3%, n = 686/908) had comorbid conditions; 47.8%, (n = 414/908) associated with chest and respiratory symptoms. Participant consulting behaviour significantly increased in the 3-month period following questionnaire completion compared with the previous 3-month period (p = .002), indicating questionnaires impacted upon consulting behaviour. Symptomatic non-consulters were predominantly younger, employed, with higher multiple deprivation scores than their GP practice mean. Of symptomatic non-consulters, 30% (18/61) consulted ≤1 month post-questionnaire, with comorbidities subsequently diagnosed for five participants. Interviews (n = 39) indicated three overarching differences between the views of consulting and non-consulting participants: concern over wasting their own as well as GP time; high tolerance threshold for symptoms; a greater tendency to self-manage symptoms.

**Conclusions:**

This first study to examine symptoms and consulting behaviour amongst a primary care population at ‘high- risk’ of lung cancer, found symptomatic patients who rarely consult GPs, might respond to a targeted symptom elicitation intervention. Such GP-based interventions may promote early diagnosis of lung cancer or other comorbidities, without burdening already pressurised services.

## Introduction

Lung cancer is the second most common cancer worldwide with 43,500 new diagnoses per year in the UK, 410,00 in Europe and 1.83m worldwide[1], and has the lowest survival rate of all cancer sites [2]. The mean doubling time for lung cancer is 125 days, but may be as rapid as 7.5 days, with two-thirds diagnosed at late stage when curative options are limited[2]. One- and five-year survival rates are lower in the UK than other European countries[3,4], which may be partly related to the structure of primary care[5]. Even small improvements in timing of lung cancer diagnosis could significantly improve survival[6]. Consequently, early diagnosis of lung cancer is a priority for the National Awareness and Early Diagnosis Initiative (NAEDI) in England[7], with a national symptom awareness campaign conducted in 2012[8].

Diagnosis of lung cancer may be partly delayed by late patient presentation in primary care. This may result from extended patient appraisal intervals (time taken to recognise and interpret bodily changes) and help-seeking intervals (time taken to act on symptoms)[9,10]. In terms of the appraisal interval, evidence indicates patients often either fail to recognise early symptoms as potentially indicative of cancer[11,12], or else normalise them by attributing them to aging processes, lack of fitness or comorbidities[13,14]. Reasons for longer help-seeking intervals include: fear of consultation; gender differences (e.g. men less ready to seek help than women); and need for ‘sanctioning’ by others[13]. Long-term smokers, those with COPD and/or those living alone are at particular risk of taking longer to consult with symptoms of lung cancer[15]. Once patients consult General Practitioners (GPs), they may not report all their symptoms or describe them in relation to everyday experiences rather than as possible signs of ill health[16]. Timely diagnosis also relies upon the skill of clinicians to elicit symptom history in consultations; their knowledge levels and attitudes, and; access to and organisation of health care[17,18]. Lung cancer has been classified as ‘harder to suspect’ than most cancers[19], while GPs encounter few patients presenting with new lung cancers each year, giving relatively little experience in diagnosis[2].

The aim of this study was to explore the help-seeking behaviour of patients at ‘high-risk’ of lung cancer, who had symptoms indicative of lung cancer, and to better understand barriers and faciliators to help-seeking amongst symptomatic patients who rarely consut GPs. Better understanding of help seeking behaviours amongst this group will facilitate the development of appropriate interventions to target individuals most at risk of lung cancer without burdening already pressurised services.

## Methods

### Sample and Data Collection

Eight GP practices from three counties in south England participated in the study, and identified individuals at ‘high-risk’ of developing lung cancer (>50 years old with smoking history within previous 10 years) from practice lists. Practices mailed potential participants a 10-page version of the IPCARD (**I**dentifying Symptom **P**redictors of **C**hest **a**nd **R**espiratory **D**isease) questionnaire, previously developed by members of the research team[20,21]. IPCARD asks individuals about the presence, severity, progression and chronicity of nine symptoms often reported by patients recently diagnosed with lung cancer[20,22]: tiredness; breathing changes; chest and upper body aches; cough; coughing up blood; non-menopausal sweats; ongoing voice changes; unintentional weight loss; and noticeably more chest infections over a 12 month period. Questionnaires also included socio-demographic questions. Data collection took place between June 2012 and January 2013. Participants’ consulting behaviours 12 months pre and post the date of questionnaire completion were extracted from electronic records at GP practices and recorded using standardised data extraction forms.

Respondents (n = 38) representing different categories of self-reported symptom profiles (symptom combinations, chronicity and severity), socio-demographic characteristics, smoking status and self-reported GP consulting behaviour over the previous 12 months were purposively sampled for semi-structured interviews. One researcher (EJ) conducted interviews, exploring help-seeking intentions and factors promoting or inhibiting help-seeking behaviour.

### Ethics

Ethical approval for the study was secured from the National Research Ethics Service (NRES) Committee South Central-Southampton A on 20/05/2012 (12/SC/0049). Completed questionnaires returned to the research team (n = 1172) implied consent for their responses to be included in the study. Separate consent forms, sent with study invitation, were signed by participants to consent for medical records review (n = 908). Further separate written consent was given prior to interviews (n = 38). This procedure received approval from the above ethics committee.

### Statistical Analysis

Data from questionnaires and clinical notes were initially entered into the same SPSS database, and then exported to Stata 13.1 for analysis. Descriptive and inferential statistics were used to explore variables and relationships between variables. Paired sample t-tests were used to compare the mean number of consultations for symptoms indicative of lung cancer for the 12 months and three months pre- and post each participant’s completion of the questionnaire, and 95% confidence intervals were calculated around the difference in proportion of those attending GP consultations for the same periods[23].

Negative binomial regression was used to model GP visits in the year post-questionnaire and to identify the variables most strongly associated with number of GP visits. GP visits prior to the questionnaire, total number of symptoms, number of comorbidities, age group, gender and site were a priori included in the model. Remaining variables (e.g. employment, education and domestic status) were included or excluded in the model based on size of incidence rate ratios (IRRs) and associated p-value. Statistical analysis was conducted by SE, LB and RW.

### Qualitative Analysis

Interviews were transcribed verbatim and analysed for themes using the computer programme NVivo 10 to facilitate thematic content analysis[24]. Coding was conducted by three experienced qualitative researchers (EJ, LB, RW). Each researcher independently coded one interview and discussed their findings with the full research group to agree upon emerging themes. Thereafter, regular two weekly discussions were conducted between the three researchers to review the development of the thematic framework and ensure analytical rigour. CR also contributed to later stages of analysis.

## Results

Of 4622 individuals identified as being at high-risk of developing lung cancer and invited to participate in the survey, 1172 (25.3%) completed and returned the questionnaire (response rates varied across practices: 19%-29%). Of these, clinical note reviews were completed on 908 respondents (77.5%). Table 1 indicates the characteristics of respondents. There was evidence of association between age group and participation (X^2^_(3)_ = 20.4, p < .001), which appears, at least in part, to be explained by a lower participation rate in the age 50–59 year old group. Participation was also independently associated with levels of social deprivation, with those in the most socially deprived quintile least likely to participate compared to other quintiles (X^2^_(4)_ = 158.9, p < .001). There were also significant variations in participation between practices (X^2^_(7)_ = 21.6, p = .011).

**Table 1:** Respondent characteristics from questionnaire (n = 1172) and clinical notes review (n = 908).

Characteristic		Eligible Patients (n = 4622)	Non-responders (n = 3449, 74.6)			Responders (n = 1172,25.3%)		Participants not consulted GP for 12 months (n = 126, 13.8%)		Symptomatic participants not consulted GP for 12 months (n = 61, 6.7%)		Symptomatic participants with no comorbidities and not consulted GP for 12 months (n = 42, 4.7%)	
		(n =)	(n =)		(%)	(n =)	(%)	(n =)	(%)	(n =)	(%)	(n =)	(%)
**Gender**	**Male**	2704	2035	75.2		669	24.7	70	14.0	35	7.0	23	4.6
	**Female**	1917	1414	73.7		503	26.2	56	14.2	26	6.6	20	5.1
	**Chi**^**2**^ **(p =)** ^a^		X^2^(2) = 1.669, p = 0.434					X^2^ = .930(1), p = .503		X^2^ = .468(1), p = .468		X^2^(1) = .132, p = .417	
**Age group**	**50–59**	1647	1287	78.1		360	21.9	61	22.8	33	12.4	25	9.4
	**60–69**	1615	1167	72.3		448	27.7	39	11.8	16	4.8	12	3.6
	**70–79**	945	682	72.1		263	27.8	18	9.3	8	4.1	2	1.0
	**80+**	415	314	75.7		101	24.3	8	11.4	4	5.7	4	5.7
	**Chi**^**2**^ **(p =)** ^a^					X^2^(3) = 20.356, ≤.001		X^2^ = 21.315(3), p≤.001		X^2^ = 16.714(3), p≤.001		X^2^ = 18.519(3), p≤.001	
**Index of multiple deprivation**	**1 –Least deprived**	749	545	72.8		204	27.2	13	19.7	13	8	5	7.7
	**2**	977	720	74.0		257	26.0	20	10.4	12	6.3	7	3.7
	**3**	1537	1163	75.1		384	24.9	41	14.5	20	7.1	14	4.9
	**4**	1036	785	75.8		251	24.2	28	14.6	11	5.8	6	3.1
	**5 –Most deprived**	313	237	75.8		76	24.2	24	14.7	5	7.6	11	6.7
	**Chi**^**2**^ **(p =)** ^a^					X^2^ = (4) = 158.873p≤.001		X^2^ = 3.990(4), p = .407		X^2^ = .854(4), p = .931		X^2^ = 4.184(4), p = .382	
**GP practice**	**Site 1**	442	346	78.6		95	21.4	17	20	12	14.1	8	9.4
	**Site 2**	459	329	71.7		130	28..3	8	9.0	1	1.1	1	1.1
	**Site 3**	679	501	73.8		178	26.2	25	17.9	14	10.0	10	7.1
	**Site 4**	745	555	74.5		190	25.5	14	9.7	5	3.5	3	2.1
	**Site 5**	693	500	72.2		193	27.8	26	17.4	11	7.4	8	5.4
	**Site 6**	166	135	81..3		31	18.7	4	16.7	3	12.5	3	12.5
	**Site 7**	884	687	77.7		197	22.3	18	11.5	7	4.5	5	3.2
	**Site 8**	554	396	71.5		158	28.5	14	13.1	8	7.5	5	4.8
	**Chi**^**2**^ **(p =)** ^a^	-	-	-		X^2^(7) = p = .011		X^2^(7) = 10.788, p = .148		X^2^(7) = 19.208, p = .008		X^2^(7) = 14.641, p = .041	
**Current smoker**	**Yes**					370		46	16.2	28	9.9	22	7.8
	**No**					782	-	79	13.2	33	5.5	20	3.3
	**Chi**^**2**^ **(p =)** ^a^					-		X^2^(2) = 1.795, p = .408		X^2^(2) = 6.597, p = .037		X^2^(2) = 9.085, p = .011	
**Ethnicity**	**White**	-	-	-		1088	-	116	14.0	57	6.9	40	4.8
	**Mixed**	-	-	-		8	-	0	0.0	0	0	0	0
	**Black/ Black British**	-	-	-		8	-	1	20.0	1	20	0	0
	**Asian/ British Asian**	-	-	-		9	-	1	12.5	0	0	0	0
	**Chinese**	-	-	-		1	-	1	100	1	100	1	100
	**Other**	-	-	-		5	-	0	0	0	0	0	0
	**Chi**^**2**^ **(p =)** ^a^	-	-	-		-	-	X^2^ = 8.140, p = .149		X^2^ = 16.218, p = .006		X^2^(5) = 20.995, p = .001	
**Domestic background**	**Married**	-	-	-		670	-	78	15.2	39	7.6	29	5.7
	**Single**	-	-	-		70	-	6	11.1	3	5.6	2	3.7
	**Divorced/ separated**	-	-	-		169	-	18	13.8	10	7.7	6	4.6
	**Widowed**	-	-	-		116	-	10	11.5	5	5.8	3	3.5
	**Living with partner**	-	-	-		76	-	11	19.0	3	5.3	2	3.5
	**Chi**^**2**^ **(p =)** ^a^	-	-	-			-	X^2^(5) = 3.175, p = ..673		X^2^(5) = 1.360, p = .929		X^2^(5) = 1.642, p = .896	
**Highest qualification**	**None**	-	-	-		291	-	20	9.4	8	3.8	2	0.9
	**GCSE/ O-Level**	-	-	-		259	-	32	15.9	16	8.0	11	5.5
	**A-Level**	-	-	-		106	-	14	16.1	5	5.7	4	4.6
	**Degree**	-	-	-		162	-	18	15.0	11	9.2	9	7.6
	**MA, PhD**	-	-	-		34	-	8	25.0	4	12.9	3	9.7
	**Vocational qualification**	-	-	-		198	-	25	15.2	12	7.3	9	5.5
	**Chi**^**2**^ **(p =)** ^a^	-	-	-			-	X^2^(5) = 7.880, p = .163		X^2^(5) = 6.584, p = .253		X^2^(5) = 11.139, p = ..049	
**Employment status**	**F/T employment**	-	-	-		256	-	44	22.3	24	12.2	18	9.2
	**P/T employment**	-	-	-		104	-	16	21.1	8	10.5	7	9.2
	**Voluntary work**	-	-	-		5	-	0	0	0	0	0	0
	**Unemployed**	-	-	-		28	-	0	0	0	0	0	0
	**Disabled**	-	-	-		27	-	0	0	0	0	0	0
	**Home-maker**	-	-	-		20	-	1	8.3	1	8.3	1	8.3
	**Retired**	-	-	-		689	-	61	11.4	27	5.1	17	3.2
	**Chi**^**2**^ **(p =)** ^a^	-	-	-			-	X^2^(8) = 25.760, p = .001		X^2^(8) = 17.234, p = .028		X^2^(8) = 18.603, p = .017	

Note

a = associated trend against all respondents

During the study period, three participants were diagnosed with lung cancer/mesothelioma, within a range of 4 weeks–11 months post-completion of questionnaire. Each of the diagnosed individuals had one or more comorbidities (i.e. asthma, hypertension, cardiovascular disease) and all reported three or more symptoms in their questionnaire, but which were not specifically referred to in their notes. The patients were not being investigated for potential lung cancer at questionnaire completion,and two died within eight and five months respectively from diagnosis.

### Symptom and Comorbidity Prevalence

A high prevalence of symptoms associated with lung cancer was reported. As Table 2 shows, 53.6% (629/1172) of all respondents reported experiencing at least one and 35% (n = 411) two or more of 9 symptoms potentially indicative of lung cancer within the previous three months. Table 2 also shows the percentage of participants who reported each of the symptoms and their chronicity. Almost a third (31.8%) of respondents reported tiredness: for 25.4% (n = 287/1172) this was experienced in combination with other symptoms. Other prevalent symptoms included breathing changes (28.3%, n = 323), increased chest infections over the previous year (24.9%, n = 292), chest aches/pain (17.3%, n = 192) and cough (13.9%, n = 161). Over a third (37.4%, n = 439) of respondents reported having first experienced at least one symptom >12 months previously (Table 2).

**Table 2:** Symptom prevalence and chronicity reported in the questionnaire (n = 1172).

Symptoms indicative of lung cancer		% of patients reporting each Symptom in the questionnaire	% of patients reporting symptoms in combination with other symptoms	% patients reporting chronicity of symptoms		
				≤ 3 months	4–12 months	>12 months
**1**	**Tiredness**	31.8% (n = 351)	25.4% (n = 287)	13.3% (n = 47)	29.3% (n = 103)	57.3% (n = 201)
**2**	**Breathing changes**	28.3% (n = 323)	23.2% (n = 265)	8.6% (n = 28)	18.6% (n = 60)	72.7% (n = 235)
**3**	**Chest and upper body aches, pain or discomfort**	17.3% (n = 192)	15.1% (n = 168)	9.8% (n = 19)	16.6% (n = 32)	73.4% (n = 141)
**4**	**Cough**	13.9% (n = 161)	8.9% (n = 104)	19.8% (n = 32)	22.3% (n = 36)	57.8% (n = 93)
**5**	**Coughing up blood**	0.1% (n = 1)	0.1% (n = 1)	0.0% (n = 0)	100% (n = 1)	0.0% (n = 0)
**6**	**Non-menopausal hot or cold sweats**	15.7% (n = 184)	13.0% (n = 149)	10.3% (n = 19)	19.5% (n = 36)	70.1% (n = 129)
**7**	**Noticeably more chest infections within the previous 12 months**	24.9% (n = 292)	14.5% (n = 170)	-	-	-
**8**	**Unintentional weight loss within the previous 12 months**	14.2% (n = 165)	8.9% (n = 104)	-	-	-
**9**	**Ongoing voice changes within the previous 12 months**	10.2% (n = 120)	9.0% (n = 103)	-	-	-
**Total number of patients reporting symptoms**		**53.6%, (n = 629)**^1^	**35.1% (n = 411)**^1^	**9.3% (109)**^1^	**17.4% (205)**^1^	**37.4% (439)**^1^

Note

1 = Totals are not the sum of all respondents/consultations within the column as many respondents consulted for more than one symptom

Of respondents included in the clinical notes review, 77.3% (n = 686/908) were found to have at least one comorbidity, 35% (n = 313/908) to have two or more. Many participants (47.8%, n = 414/908) were living with comorbidities that might impact on their respiratory function, most commonly Chronic Obstructive Airways Disease (COPD) (n = 89/908, 9.8%), asthma (n = 71/908, 7.8%) and cardiac disease (n = 79/908, 8.7%). Using Chi^2^, a positive association was found between those participants reporting symptoms in the questionnaire and those found to be living with comorbidities (X_(1)_ = 15.8, p < .001). These findings indicate symptoms associated with lung cancer are very common amongst this group of high-risk patients and are likely often? caused by other common conditions.

### GP Consulting Behaviour

Note reviews found that 216 respondents collectively consulted their GP for potential lung cancer symptoms on a total of 355 occasions in the 12 months pre-questionnaire, compared with 247 respondents consulting on 415 occasions in the 12 months following the questionnaire: an increase of 14.4%. Using McNemar’s difference in proportions, we found an increase of 3.4% (95% CI: 0.0, 6.8) in overall consultations between the 12 months pre-questionnaire compared with post-questionnaire, and a significant increase of 4.2% (95% CI: 1.8, 6.5) in the number of consultations between the three months pre- and post-questionnaire (Table 3). Almost half the participants (45.4%, 413/908) for whom we have consultation data reported symptoms in questionaiires for which they did not consult the GP.

**Table 3:** GP consultations for symptoms pre- and post- completion of the IPCARD survey (n = 908).

Symptoms presented to GPs	12 months prior to questionnaireN = respondents (n = GP visits)	12 months following questionnaireN = respondents (n = GP visits)	Difference in total % of participants visiting GP (95% CI)	Difference in mean GP visitsSignificancet(df) =, p =)	3 months prior to questionnaireN = respondents (n = GP visits)	3 months following questionnaireN = respondents (n = GP visits)	Difference in total % of participants visiting GP (95% CI)	Difference in mean GP visitsSignificancet(df) =, p =)
**Tiredness**	17 (27)	30 (33)	+76.4%	t(908) = .500, p = .617	7 (9)	12 (13)	+71.4%	t(908) = -.784,p = .433
**Breathing changes**	62 (77)	73 (100)	+17.7%	t(908) = -085,p = .932	15 (18)	24 (30)	+60.0%	t(908) = -1.908,p = .057
**Chest infection**	64 (76)	94 (124)	+46.8%	t(908) = 2.765, p = .006	15 (18)	33 (37)	+94.4%	t(908) = -3.078,p = .002
**Chest pain**	28 (32)	33 (37)	+17.8%	t(908) = .308,p = .758	7 (8)	9 (10)	+28.5%	t(908) = -.625,p = .532
**Cough**	123 (166)	139 (192)	+13.0%	t(908) = 1.320,p = .196	32 (46)	50 (55)	+56.5%	t(908) = -2.034,p = .042
**Coughing up blood**	5 (6)	6 (6)	+20%	t(908) = -838, p = .402	2 (2)	2 (2)	0%	t.(908) = 1.000 p = 1.000
**Non-menopausal sweating**	9 (9)	9 (10)	+0%	t(908) = .430, p = .667	3 (3)	2 (3)	-50.0%	t.(908) = 1.000 p = 1.000
**Unintentional weight loss**	7 (10)	10 (11)	+42.8%	t(908) = 1.381, p = .168	2 (2)	3 (3)	+50%	t(908) = -.447, p = .655
**Voice changes**	6 (6)	4 (5)	+33%	t(908) = .442, p = .658	0 (0)	1 (1)	+100%	t(908) = -1.000, p = 318
**Total GP consultations**	**216 (355)**^1^	**247 (415)**^1^	**+3.4%(CI: 0.0, 6.8)**	**-**	**55 (67)**^1^	**93 (113)**^1^	**+4.2%(CI: 1.8, 6.5)**	**-**
**Mean GP consultations**	**0.30(SD = .884)**	**0.46(SD = 968)**	**-**	**t(907) = -1.951, p = .051**	**0.0738(SD = 0.33871)**	**0.1244 (SD = 0.40523)**	**-**	**t(907) = -3.074,p = .002**

Note

1 –Totals are not the sum of all consultations within the column as many individual consultations were for more than one symptom

Paired sample t-tests compared the mean number of consultations for each of these symptoms for the 12 months and three months pre- and post completion of the questionnaire. Analysis found that only consultations for chest infections increased significantly over the 12 month period (p = .006): the overall increase of consultations for symptoms potentially indicative of lung cancer just missed statistical significance (p = .051). However, a significant increase was found in the mean number of GP consultations for symptoms in the three months following the completion of the questionnaire, amongst this sample (M = 0.1244, SD = 0.40523), compared with the three month period before participants received the questionnaire (M = 0.0727, SD 0.32398); p = .002), with significant increases for both chest infections (p = .002) and cough (p = .042). Moreover, the proportional increases for consulting behaviour in the 3-month period following receipt of the questionnaire for some symptoms (i.e. chest infections: 94.4% (p = .002); cough: 56.5% (p = .042); chest pain: 28.5% (p = .532), and; breathing changes: 60% (p = .057)) were greater than the proportional increases for these symptoms in the overall 12 month period. This finding suggests that completing the IPCARD questionnaire may have encouraged participants to increase their consulting behaviour, but that most of this increase occurred in the short term.

Negative binomial regression identified those variables most strongly associated with post-questionnaire GP visits. In order of inclusion, the variables completing the model were employment status, domestic status (married/single etc.), highest qualification attained and severity of breathlessness. Squared terms for pre-questionnaire visits, symptoms and comorbidities were also tested (Table 4). Higher numbers of reported symptoms (p < .001), increased total number of comorbidities (p < .001), and increased pre-questionnaire visits (p < .001) were all independently associated with increased post-questionnaire GP visits. Of the symptoms, only the reported severity of breathlessness was associated with GP visits, and was highly correlated with the total number of symptoms reported by participants, meaning those with more reported symptoms had more severe breathing changes. There was noticeable variation across sites.

**Table 4:** Negative binomial Regression analysis: Participant characteristics and consultation behaviour.

Variable*	IRR	95% CI	p-value
**Pre-questionnaire GP visits**			
*Linear term*	1.084	1.064–1.104	< .0005
*Squared term*	0.999	0.998–0.999	< .0005
**Total symptoms**	1.094	1.043–1.147	< .0005
**Number of comorbidities**	1.151	1.089–1.216	< .0005
**Gender** (ref. male)	1.082	0.955–1.226	.215
**Age group** (ref. 50–59 years)			
*60–69*	1.128	0.944–1.347	.185
*70–79*	1.315	1.047–1.653	.019
*80+*	1.442	1.081–1.924	.013
**Employment** (ref. full-time)			
*Part-time*	0.686	0.532–0.884	.004
*Retired*	0.833	0.687–1.011	.065
*Other*	0.842	0.650–1.090	.192
**Domestic** (ref. married)			
*Single*	1.082	0.837–1.398	.547
*Divorced/separated*	0.959	0.805–1.144	.644
*Widowed*	1.298	1.060–1.589	.012
*Living with partner*	1.115	0.877–1.416	.374
**Highest qualification** (ref. none)			
*GCSE/O-level*	0.933	0.786–1.109	.432
*A-level*	0.786	0.629–0.982	.034
*Degree*	0.924	0.762–1.119	.418
*MA*, *PhD*	1.076	0.788–1.469	.646
*Vocational*	0.949	0.796–1.130	.555
**Severity breathlessness**	0.965	0.937–0.993	.015

*Site not shown; IRRs ranged from 1.007 (95% CI [0.769, 1.320]) to 1.549 (95% CI [1.179, 2.035]). Parameter α (to model additional dispersion in negative binomial model) estimated as 0.289 (95% CI [0.229, 0.365]).

### Symptomatic Non-Consulters

Of all participants whose notes were reviewed, 126/908 (13.8%) were found not to have attended their GP practice for any reason for 12 months prior to the survey (Table 2 shows socio-demographic characteristics of these participants). For many this was unsurprising, as they had reported no symptoms. However, 61/126 non-attenders (48.4%) had reported symptoms in the questionnaire, and of these 42/61 (68.8%) had no diagnosed comorbidities to which experienced symptoms were potentially attributable (Table 1). Therefore, a group of non-attenders with potential lung cancer symptoms were identified (61/908; 6.7% of respondents), most of whom did not have a diagnosis of chest or respiratory disease that might explain symptoms (42/908% of respondents).

**Fig 1:**
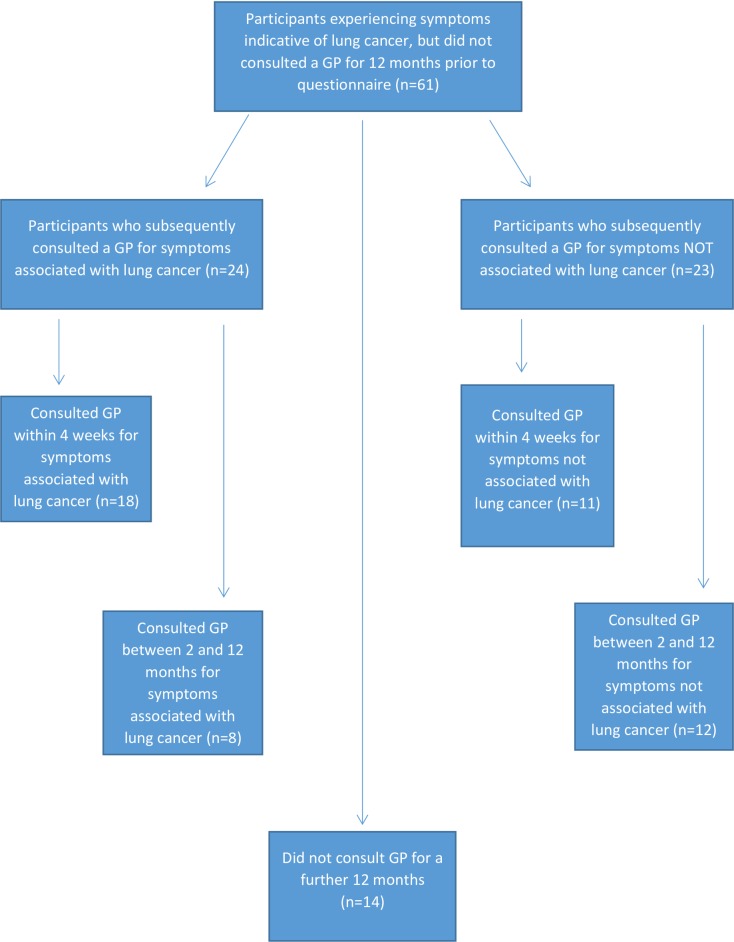
Post questionnaire help-seeking behaviour of participants with symptoms associated with lung cancer who had not consulted a GP for 12 months.

Symptomatic non-consulters were predominantly male (35/61, younger (mean/median age: 61.8/59 years; range 50–93), employed (32/61) and had index of multiple deprivation (IMD) scores lower than their GP practice average. Within one month of completion of the IPCARD questionnaire, 29% (18/61 29%) symptomatic participants who had not consulted their GP for 12 months subsequently consulted their GPs for their symptoms. Following GP consultation, six were treated for chest infections (all of whom attended for cough), nine were given health checks and/or lifestyle counselling (including smoking cessation advice), and five had previously unknown comorbidities diagnosed (e.g. COPD, emphysema, asthma, hypertension and depression) (Table 5). Eight symptomatic non-consulters consulted for symptoms potentially indicative of lung cancer within 2–12 months of completing the questionnaire (see Fig 1). Of the 37 participants who did not consult at all for IPCARD symptoms, 11 consulted within four weeks for reasons other than those within the IPCARD survey (i.e. urine infection, cholesterol check, leg ulcer), 12 consulted some months later for a range of similar reasons, and 14 did not consult for at least a further 12 months.

**Table 5:** Data relating to symptomatic, non-consulting participants who visited GPs <1 month post survey completion (n = 16).

No.	Age	Gender	Current smoker	Symptoms reported in questionnaire	No. of days to consultation	Reason for seeking help	Investigations	Outcome
**1**	52	Male	Yes	Cough >12m, sob> 12m, fatigue> 12m,	27	Cough	Bloods, CXR (lung changes)	**COPD Diagnosed**, referred for SCA
**2**	61	Male	Yes	SoB, Fatigue, voice changes	26	Cough, SoB	Bloods,CXR (lung changes)	**Asthma Diagnosed**, referred for SCA
**3**	58	Female	Yes	Tiredness 3m	11	Cough	Health Check	Referred for SCA
**4**	56	Female	Yes	Wt loss	17	Wt loss.	Bloods–NAD	Health check; referred for SCA
**5**	57	Male	Yes	Cough 4-12m	19	Cough	Bloods–NAD	Referred for SCA
**6**	63	Male	Yes	C/P >12m	23	Cough	-	Treated for chest infection Abx & follow up
**7**	55	Male	No	Cough– 3m	21	Cough	-	Treated for chest infection Abx; Health Check
**8**	56	Female	Yes	Cough >12m	10	Cough	-	Treated for chest infection Referred for SCA
**9**	68	Male	No	Cough 4-12m, Increasing ch/infections; voice changes	5	Cough, Ch/inf	CXR (lung changes)	**Emphysema diagnosed**
**10**	54	Male	No	C/P 4-12m,	24	C/P, breathing changes	Bloods–NAD	Treated for chest infection ABx & followed up
**11**	56	Male	No	C/P>12m, cough >12m, sweats >12m, Weight loss	27	Slight wheeze	Bloods–NAD	Watch &Wait (WW); no follow-up recorded.
**12**	53	Male	Yes	Breathing changes>12m	17	Breathing changes	CXR–NAD	**Hypertension Diagnosed** & Obesity. Refered for lifestyle counselling/ SCA
**13**	83	Female	No	Tiredness 3m, voice changes	23	Tiredness	Bloods–NAD	**Depression Diagnosed** & insomnia. Refered for counselling
**14**	55	Female	Yes	Tiredness >12m	14	Fatigue	Bloods–NAD	Health check; referred for SCA
**15**	57	Male	Yes	Weight loss	18	Weight loss	CXR–NAD	Refered for lifestyle counselling/ SCA
**16**	65	Female	Yes	Tiredness >12m	15	Tiredness	Health check	Refered for SCA
**17**	67	Female	Yes	TBA	23	Cough	-	Treated for chest infection Referred for SCA
**18**	74	Male	No	TBA	16	Cough	-	Treated for chest infection

Note: Abx: antibiotics; Ch/inf: chest infection; CP: chest pain; COPD: chronic obstructive pulmonary disease; CXR: chest X-ray; NAD: no abnormalities discovered; SCA: smoking cessation advice; SoB: shortness of breath.

### Patient Interviews

Interviews (n = 38) were conducted to explain the help-seeking behaviour of participants and the issues that had most impact upon help-seeking (see Table 5). There were themes that were common to all participants, but we also compared the views and experiences of consulting and non-consulting participants. Socio-demographic characteristics are provided for both groups in Table 6.

**Table 6:** Characteristics of interview participants (n = 38).

**Characteristic**		**Responders (n = 38)**		**Those not seeing GP in last 12 months (n-7)**	
		(n =)	(%)	N	(%)
**Gender**	Male	23	61	5	71
	Female	15	40	2	29
**Age group**	50–59	19	50	4	58
	60–69	8	21	1	14
	70–75	6	16	1	14
	75+	5	13	1	14
**Index of multiple deprivation (rank)**	1 –Most deprived	7	18	1	14
	2	8	21	2	29
	3	13	34	1	14
	4	7	18	0	0
	5 –Least deprived	3	8	3	29
**Ethnicity**	White	37	97	7	100
	Black/ Black British	1	3	0	0
**Domestic background**	Married	15	40	4	57
	Single	5	13	2	28
	Divorced/separated	8	21	0	0
	Widowed	5	13	1	14
	Living with partner	5	13	0	0
**Highest qualification**	None	9	24	2	28
	GCSE/ O-Level	13	34	3	44
	A-Level	5	13	0	0
	Degree	2	6	0	0
	MA, PhD	1	3	0	0
	Vocational qualification	7	18	2	28
	Missing	1	3	1	14
**Employment status**	F/T employment	11	28	3	44
	P/T employment	3	8	1	14
	Voluntary work	1	3	0	0
	Unemployed	3	8	0	0
	Unpaid leave	1	3	0	0
	Disabled	2	6	0	0
	Retired	17	45	2	44
**Smoking status**	Current smoker	24	63	2	29
	Past smoker	14	37	5	71

Participants generally revealed a ‘wait and see’ attitude towards most symptoms. With age, some participants appeared to become more accepting of illness, and in particular tiredness and breathlessness. Often symptoms would worsen or persist for months before participants contacted their GP. Participants indicated they would more likely seek help if they experienced recurrence of specific, previously experienced worrisome symptoms; painful symptoms; unfamiliar symptoms; and recent changes in symptoms. Specific symptoms that had motivated greater help seeking were: severe breathing difficulties; ‘horrible’ chest pains; long-lasting chest infections; cough; haemoptysis and significant weight loss in the short-term. A degree of severity was often described as necessary to trigger help seeking.

Even when symptoms were worrisome, however, some participants would hesitate to contact their GP., Female current smokers In particular experienced feelings of guilt for symptoms perceived as ‘self-inflicted’. Men especially would delay until encouraged by friends/family to consult GPs. There were fears amongst both men and women of wasting GPs’ time. Issues also concerned difficulty accessing appointments, especially if in work, and time wasted in waiting rooms. Even when consulting GPs, participants indicated they did not always report true smoking habits or symptoms.

Three over-arching themes emerged in which there were differences between the views of consulters and non-consulters: not wanting to waste time; appraising symptoms; and attitudes to help-seeking.

#### Not wanting to waste time

Amongst those individuals who had not sought help in 12 months, expressed concerns about wasting their GP’s time were shorter and accompanied by prolonged accounts of the patient not wanting to waste their *own* time. Talk of patient's wasted time occurred in 4/7 interviews with people who had not consulted in the last year and in no interviews with regular consulters.

‘[I] hate going to the doctors when you’ve got to sit for an hour or two hours waiting….I’ve always been a very, very busy person… For me I just don’t like the process and I know they are very, very busy people so I don’t want to waste their time either. … [I once got] to the stage where I thought right OK I’ll go and waste a couple of days going backwards and forwards to specialists and doctors and all the rest of it. (05/575)

Moreover, non-consulters were the only participants who distanced themselves from consulting behaviours of patients they perceived did waste GP time. Male non-consulters were also more likely than male consulters to refer to the idea that men are not comfortable seeking helping, and suggested that their family (especially partners) or friends had encouraged them to consult their GP for a problem.

I try to heal myself as much as I can until the wife gets me and points me in the right direction and tells me to get down there and I don’t tend to argue with the wife because she’s always right….I mean I’ve been married about 38 years now, so she knows me quite well and she takes a firm stance at certain stages. And I think she knows some of the symptoms better than I do and to be honest with you, she tells me that I’m going to the doctors—and I go. (07/037)

#### Appraising symptoms

While a degree of severity was often described as necessary to trigger help seeking, non-consulters indicated they had a higher tolerance threshold to symptoms, giving the least rich accounts of symptoms that triggered them to seek help.

How would I make that judgement call? If I suddenly realised hang on this isn’t getting better it needs, I need antibiotics or I need something needs to be done then I will go to the GP but if it’s just every day stuff I don’t go so it would have to be pretty severe for me to make an appointment (09/059)

With a combined low threshold for ‘wasting’ their own time at practices, and a high threshold for tolerating worrisome symptoms, there is greater likelihood for these patients to not seek help.

#### Attitudes to help-seeking

Both consulters and non-consulters downgraded symptoms they experienced as minor or not worthy of GP consideration, particularly compared with people who are ‘much more ill’ or with greater ‘need’. However, this was more evident amongst non-consulting participants.

*I just feel like I’m wasting their time and there are people that need to be there more than I do*. *So I don’t go*. (09/059)

Once participants decided their symptoms warranted intervention, non-consulters were more likely to attempt to self-manage their condition and if that was not successful, to go and see their GP. Non-consulters were more likely to seek information from the internet and books and gave the impression they were more empowered to deal with problems themselves, more self-sufficient.

## Discussion

### Summary of Findings

This mixed methods study incorporated a patient symptom assessment survey, clinical notes review and patient interviews. Triangulation of data from these different sources provides significant new insights into how approaches to raising awareness and early detection amongst primary care populations should be targeted upon those at high-risk of lung cancer. In particular, there is a clinically relevant group of patients who rarely or never consult their GP, who may also have few diagnosed comorbidities, and yet may experience worrisome symptoms. This group were predominantly male, younger, smokers and had IMD scores lower than the practice average; the same group statistically least likely to respond to the questionnaire. As this study has shown, even if experienced symptoms are not signs of lung cancer, they may be indicative of other commorbidites such as COPD, asthma or emphysema. Targeting these individuals within primary care with interventions designed to facilitate earlier diagnosis may prove effective and resource-efficient, and while the focus may be lung cancer, other comorbidities may also be discovered.

### Comparisons with Other Studies and Interpretation of Our Findings

Our survey findings identified a high ‘baseline’ level of reported symptoms associated with lung cancer within this ‘high-risk’ population sample. While previous evidence found 11% of a general population sample experienced a possible cancer symptom within the previous three months[25], our study found 54% of high-risk individuals reporting at least one symptom in this period. Even excluding participants experiencing tiredness only, which may be thought ubiquitous, 40% of all survey respondents experienced one or more potential lung cancer symptoms. A study exploring symptoms predictive of lung cancer amongst patients already referred to secondary care similarly found multiple synchronous symptoms, with symptoms other than haemoptysis unable to differentiate lung cancer from other diagnoses[26].

Research has shown that individuals with cancer present more frequently in primary care with non-specific but suggestive symptoms of lung cancer than matched controls[2,27], while our regression analysis found that no single symptom predicted increased GP consultations amongst an at-risk primary care population. The CAPER studies and QCancer algorithums have provided an evolving set of risk prediction models for cancer types, including lung cancer[27,28], and for the risk of cancer overall[29,30]. Nevertheless, of a sample of patients who subsequently developed lung cancer, between 17%-34% of symptoms presented in the previous 24 months were not caused by the cancer[31]. Our clinical notes review also identified high levels of comorbidities affecting respiratory function (i.e. COPD), which previous research has found frequently precedes lung cancer[32]. As Bowen et al. have shown, symptoms associated with these other diseases are difficult to distinguish from those of lung cancer[33], and both patients and GPs may attribute new or worsening symptoms to existing comorbidities[16]. Thus, the high levels of cough, breathlessness and chest infections within our sample confirm previous evidence that these symptoms alone lack specificity for lung cancer[2,27]. Our study findings therefore strengthen previous arguments that education about symptoms alone is insufficient to tackle late diagnosis[13].

Almost half the participants in this study (n = 413/908) indicated they had one or more of nine potential lung cancer symptoms for which they did not consult their GP. Previous research found individuals often delay some weeks prior to seeking help as they appraised symptoms, waiting to see how they developed, and symptoms would often need to significantly deteriorate to prompt help-seeking[34]. Interview data from our study has found that similar processes led to extended appraisal intervals even amongst participants who regularly attended their GP practice, and evidence has previously shown that many patients who die from lung cancer were already interacting with primary care for other problems prior to diagnosis[35]. Indeed, patients referred to secondary care for suspected lung cancer have been found to have similar symptom pathways whether or not they were diagnosed as such or were found not to have cancer[36]. Our study data also indicated participants consulted GPs less frequently for symptoms they deemed minor (e.g. sweats, voice changes) and for patterns of symptom onset and progression that were gradual. Indicative of this were the high number of participants who reported tiredness, unintentional weight loss and ongoing voice changes in the IPCARD questionnaire, but rarely consulted GPs specifically for these symptoms.

As in previous studies, further reasons given by interviewees in the current study for delaying GP consultation included a fatalistic perception that their condition was ‘*self-inflicted*’, that they did not want to ‘*burden*’ GPs, and may consequently think themselves unworthy of medical attention[22,37], and that men especially required ‘sanctioning’ or endorsement from within their social networks to seek medical help[12,36]. Interviewees in our study also confirmed previous evidence that primary care patients experienced difficulties booking conveniently timed appointments around work and family commitments, and feared long periods in waiting rooms[38]. However, our interview data also showed participants who had not consulted their GP for at least 12 months were particularly concerned not to waste *their own time* in GP practices, had a higher tolerance threshold for symptoms that might trigger others to consult their GPs, and were more likely to self manage symptoms and seek information from sources other than the GP practice. They might therefore be defined as ‘harder to reach’ (HtR) and of particular concern[39,40]. Of this group, 29% (n = 18) consulted their GP for symptoms indicative of lung cancer within one month of completing the symptom survey, and of these a quarter (n = 5) subsequently had comorbidities diagnosed that were previously unknown. Although for some of these patients no cause was determined for their symptoms, eight were nevertheless referred for smoking cessation advice (SCA). That these HtR participants were prepared to seek help for their symptoms after completing the survey indicates it may be a method for targeting this group; providing the opportunity for health education and interventions encouraging them to more readily consult their GP in the future.

The national ‘Be Clear on Cancer: Three-week cough’ symptom awareness campaign[8], conducted for three months (April–June) in 2012, increased consulting behaviour and facilitated a significant increase in the rate of lung cancer diagnoses in England[41]. However, the campaign was more effective amongst less deprived patients[42], and the success was accompanied by a large increase in additional workload[43,44], over which GPs had no control[45]. Thus, a more targeted approach of those groups most likely to benefit from an intervention would promise to be both effective and resource-efficient. Recent studies have also indicated the potential effectiveness of targeted, local GP-based interventions, which combine symptom awareness, education, and strategies that reduce complexity in appointment scheduling specifically for patients at-risk of lung cancer[34,45,46]. A randomised controlled trial of one such intervention is currently underway in Australia[47].

Our study findings provide further evidence for targeted interventions to facilitate timely diagnosis of lung cancer, particularly for at-risk patients who rarely consult their GP. The IPCARD questionnaire used in our study was found to be an effective tool for eliciting symptoms experienced by this patient group, despite their reluctance to consult a GP practice. Indeed, a significant overall increase in the number of consulations for symptoms identified on IPCARD by participants occurred in the three months following completion of the questionnaire, compared with the same period prior to receipt of the questionnaire. That questionnaires were sent to participants from their GP practice may also have acted to ‘sanction’ their help-seeking for these specific symptoms, and a practice-based, targeted intervention using IPCARD would give GPs greater control over additional workloads. An intervention might include rapid access routes for this group that reduce structural barriers to consultation, and education encouraging individuals to more readily report symptoms they experience to GPs in the future. While the symptomatic, non-consulting group we identified in this study represented a small proportion (7%) of the overall study sample, extrapolating this proportion across the primary care population would identify large numbers of patients at risk of lung cancer who might benefit from a targeted intervention.

### Strengths and Limitations

A key strength of the study is that data from multiple sources have been synthesised to provide new insights into how awareness and early diagnosis initiatives can be most effectively designed. Use of the IPCARD questionnaire to elicit symptom prevalence amongst a primary care population was shown feasible, requiring little work by practices to identify high-risk patients and mail-out questionnaires. IPCARD response rates were consistent across eight practices, and although low were comparable with other primary care postal surveys[48]. Despite the increase in participant consultation rates for potential lung cancer symptoms following completion of IPCARD, it is not possible to conclude definitively that any causal relationship existed. The national ‘Be Clear on Cancer’: ‘three-week cough’ campaign took place in the three months preceeding the start of this study[8]. However, there was a difference between consulting behaviour in the 3 months pre and post questionnaire completion, and for 64.1% (n = 582/908) participants, the survey was completed more than 3 months after the end of the cough campaign.

## Conclusion

This is the first study to examine symptoms and consulting behaviour in a primary care population at high-risk of lung cancer (≥50 years old with recent smoking history). Amongst this population, a small but clinically relevant group of symptomatic non-consulting individuals were identified, who despite experiencing symptoms potentially indicative of lung cancer, did not consult their GP for 12 months or more. Community, GP-based interventions targeting this population group may complement national cancer awareness campaigns to promote early diagnosis of lung cancer and other comorbidities, without creating large additional workloads to already pressurised services.
